# The Plant as Metaorganism and Research on Next-Generation Systemic Pesticides – Prospects and Challenges

**DOI:** 10.3389/fmicb.2016.01968

**Published:** 2016-12-08

**Authors:** Zisis Vryzas

**Affiliations:** Laboratory of Agricultural Pharmacology and Ecotoxicology, Department of Agricultural Development, Democritus University of ThraceOrestias, Greece

**Keywords:** soil applied pesticide, PGPR, root exudates, biological membranes, metaphysiology, rhizosphere, next-generation pesticides, nanopesticides

## Abstract

Systemic pesticides (SPs) are usually recommended for soil treatments and as seed coating agents and are taken up from the soil by involving various plant-mediated processes, physiological, and morphological attributes of the root systems. Microscopic insights and next-generation sequencing combined with bioinformatics allow us now to identify new functions and interactions of plant-associated bacteria and perceive plants as meta-organisms. Host symbiotic, rhizo-epiphytic, endophytic microorganisms and their functions on plants have not been studied yet in accordance with uptake, tanslocation and action of pesticides. Root tips exudates mediated by rhizobacteria could modify the uptake of specific pesticides while bacterial ligands and enzymes can affect metabolism and fate of pesticide within plant. Over expression of specific proteins in cell membrane can also modify pesticide influx in roots. Moreover, proteins and other membrane compartments are usually involved in pesticide modes of action and resistance development. In this article it is discussed what is known of the physiological attributes including apoplastic, symplastic, and trans-membrane transport of SPs in accordance with the intercommunication dictated by plant–microbe, cell to cell and intracellular signaling. Prospects and challenges for uptake, translocation, storage, exudation, metabolism, and action of SPs are given through the prism of new insights of plant microbiome. Interactions of soil applied pesticides with physiological processes, plant root exudates and plant microbiome are summarized to scrutinize challenges for the next-generation pesticides.

## The Plant as Metaorganism and Soil Applied Pesticides

### Structure of the Plant Microbiome

Over the last few years, considerable attention has been devoted to the concept of “plant as metaorganism.” Healthy plants host symbiotic and non-symbiotic rhizo-epiphytic and/or endophytic microorganisms that do not cause diseases but support the host nutritionally by stimulating germination and growth or help the plant to overcome biotic or abiotic stress. Therefore, plants have to be considered as metaorganisms revealing close relationships with their associated microorganisms (Berg et al., 2015). The plant microbiome consists a “second genome” that is up to 10 times more in scale than the host genome (Turner et al., 2013). The composition of the rhizosphere microbiome is dynamic, contains many more microbial cells than host cells and is influenced by multiple factors. Root microbiome is tightly related to the health of the plants and any changes in the core-microbiome composition lead to debilitative or destructive diseases as in the case of gut microbiome and human health (Kinross et al., 2011).

### Mechanisms of Action

**FIGURE 1:**
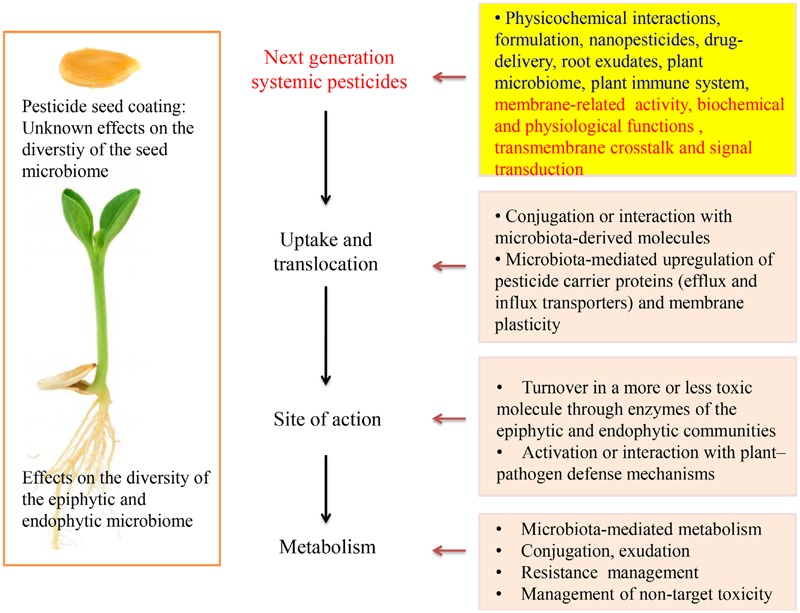
**Research challenges and perspectives in developing new systemic pesticides**.

Plant growth-promoting rhizobacteria (PGPR) and fungi (PGPF) can stimulate plant growth through the production of phytostimulators (auxins, gibberellins), increase the nutrients uptake (nitrogen fixation, phosphate solubilization), even confrere tolerance to plants against abiotic stress such as drought and salinity or by suppressing biotic stressors like plant diseases or pests (Lugtenberg and Kamilova, 2009; Pineda et al., 2010; Wang et al., 2012; Zamioudis and Pieterse, 2012). According to our studies, PGPR enhanced uptake of thiamethoxam and acibenzolar-*S*-methyl in corn and tomato plants, respectively (Myresiotis et al., 2014, 2015). During integrated control management against soilborne plant pathogens studied by our group, increased efficacy of pesticides was observed when PGPR were combined with soil applied pesticides (Myresiotis et al., 2012a). Suppression of plant diseases and tolerance against pests are often achieved through mechanisms such as the elicitation of an induced systemic resistance (ISR), production of antibiotics and lytic enzymes and competition with pathogens for nutrients and colonization sites (Kloepper et al., 2004; Van Wees et al., 2008). The development of ISR in plants depends on jasmonic acid, ethylene, or/and salicylic acid priming, which are important endogenous signaling defense regulators against pathogens and is responsible for activating the expression of pathogenesis-related genes (Buonaurio et al., 2002; Pieterse et al., 2009; Vlot et al., 2009). Recently, the role of PGPR and other beneficial microorganisms, belonging to plant microbiome, on the degradation of soil applied pesticides has been studied (Gurska et al., 2009; Myresiotis et al., 2012b; Zhou et al., 2013; Abraham and Silambarasan, 2014). While most of these studies showed that PGPR increase the degradation of some pesticides, others report that certain PGPR have no effect on biodegradation of specific pesticides. Recently, the role of endophytic bacteria on plant growth-promoting characteristics, phytoremediation of organic pollutants and other plant physiological processes is reconsidered (Barac et al., 2004; Ferrara et al., 2012; Syranidou et al., 2016). Nonetheless, the role of systemic pesticides (SPs) on endophytic microbial consortium has not yet been studied (**Figure 1**). On the other hand, endophytic bacteria usually act on host cells or stimulate biological systems by using enzymatic processes or ligands (adhesins) which are also expected to interact with SPs. Metabolism, conjugation and complex formation within plant compartments are processes that affect pesticide efficacy and fate.

### Plant Microbiome and Root Exudation

Although a single beneficial microorganism is already recommended for soil applications and management of plant diseases, information on plant microbiome suggests that microbial consortia or bespoke artificial root microbiome transfer can be more effective (Gopal et al., 2013). Moreover, current research indicates that various different volatile metabolites released by soil bacteria are capable of stimulating physiological responses to other microorganisms and plants (Wenke et al., 2010; Effmert et al., 2012; Abrudan et al., 2015; Kai et al., 2016). It is well documented that composition of plant root exudates play pivotal role in the rhizosphere microbiome (Chaparro et al., 2013). Plant roots release up to 20% of their photosynthetic fixed carbon into the soil during vegetation period and the phenomenon called rhizodeposition played an important role in chemo-attract and repellent processes (Hutsch et al., 2002; Badri and Vivanco, 2009; Jones et al., 2009). However, it has recently been observed that root exudates are ecologically relevant to plants (withstand herbivory, inhibit the growth of competing plant by allelopathy, promote the recognition of host plant by the parasitic plants and cause loss of organic compound), important for soil structure (modify the chemical and physical soil properties), and soil microflora (regulate the soil microbial community, facilitate beneficial symbioses) (Rasmann and Agrawal, 2008; Sanon et al., 2009; Doornbos et al., 2012). Additionally, root exudates trigger biofilm formation on the roots of host plants and enhance biocontrol against many pathogens (Chen et al., 2013). Mechanisms of rhizodeposition include sloughing-off root cap cells, secretion of mucilage, passive diffusion of root solutes and senescence of epidermal and cortical cells (Nguyen et al., 2009). Various root exudates such as sugars, growth regulators, amino acids, organic acids, phenolic acids, flavonoids, enzymes, fatty acids, nucleotides, tannins, steroids, terpenoids, alkaloids, polyacetylenes, phytosiderophores, and vitamines have been detected in rhizosphere (Seigler, 1996; Dakora and Phillips, 2002). The nature and diversity of root exudations is highly influenced by the plant genotype, developmental stage, a multitude of environmental factors (soil properties, temperature, pH, humidity, nutrients), rhizosphere microbiome and the application of pesticides (Bais et al., 2006; Dinelli et al., 2007; Sun et al., 2013; Lu et al., 2015). Apart from endogenous exudates, plants are capable of exuding pesticides applied to aerial part of plants (Dinelli et al., 2007). Furthermore, bioavailability, enantioselective uptake and translocation of soil applied pesticides can also be modified by different root exudates as mainly have been observed during phytoremediation studies (Lu et al., 2015; Hurtado et al., 2016). Although pesticide exudation in plants is not as extensive as in animals, large amounts of volatile pesticide or those mainly translocated through phloem can be exuded from roots (Schröder et al., 2007).

## Systematicity of Soil Applied Pesticides: Uptake and Translocation

The first classes of pesticides marketed had only contact action. However, by the 1950s many classes of SPs, which were able to enter plants by roots, stem or leaves and be translocated to other parts of the plant have been developed. Many herbicides, insecticides and fungicides have been registered for use in soil to control weed, soil-born diseases, and protect plants from herbivore pests. The standard application methods of soil pre- or post-emergence pesticides have been to be applied in farrow during planting or spray directly to the soil. Granular or liquid formulations were commonly used in the past but have been discontinued due to resistance development, environmental concerns, regulatory and cost reasons. Seed coating with fungicides and insecticides is a well-established young plants protection method form pathogens and pests. Current seed coating technology requires much less active ingredient (0.1–1.5 mg kernel^-1^ or 10–100 g ha^-1^) than the respective soil application rates (Taylor et al., 2001; Pataky et al., 2005; Girolami et al., 2009; Krupke et al., 2012). However, a single kernel contains several orders of magnitude more active ingredient than the toxicological endpoints of beneficial organisms (e.g., pollinators). Arguably, however, it is the systemic nature of soil applied pesticides and their long-lasting high concentration that made them so admissible for prophylactic applications mainly by seed coating technologies. Irrespective of their main purpose of use and their mode of action, SPs are translocated through plant and affect many physiological processes (including but not limited to their main target sites). Such collateral effects are well known in fungicides (Zhang et al., 2010; Kumar et al., 2016) insecticides (Kaufman et al., 1971; Dhungana et al., 2016) and herbicides (Fletcher et al., 1996; Cedergreen, 2008). Seed microbiome and seed-associated endophytes that might have co-evolved for millions of years have recently been associated with the establishment of plant microbiome (Johnston-Monje et al., 2016; Khalf and Raizada, 2016; Pitzschke, 2016). However, effects of seed processing and seed coating with pesticides on seed microbiome and respective colonization and establishment of plant microbiome have not been studied yet (**Figure 1**).

Systematicity of pesticides can be increased with the co-formulation with polymers (Dieckmann et al., 2010) or the use of nanomaterials. Unlike conventionally pesticide formulations, nanopesticides and targeted delivery techniques may enhance or give new biological activity to an active ingredient (**Figure 1**). Nanomaterials can cross plasma membrane, bind with cytoplasmic organelles and interfere with metabolic processes (Jia et al., 2005; Lin and Xing, 2008). Furthermore, there are several studies demonstrating nanoparticle mediated alteration of pesticide uptake and induction of genetic or cell physiological effects (Racuciu and Creanga, 2007; De La Torre-Roche et al., 2012; Hamdi et al., 2015). In addition, nanopesticides can mediate the metabolic profile in root exudates affecting indirectly the plant defense system (Zhao et al., 2016). The uptake and translocation of nanoparticle across root cells involve active and passive transport processes similar to those observed for nutrients, plant exudates, pesticide molecules, and signaling substances involved in plant defense.

Physicochemical properties of a pesticide and interaction with soil, plant microbiome, water, and chemicals surrounding the rhizosphere determine the behavior of pesticides within plant (uptake, translocation, action, detoxification, and excretion). The systemic action of most pesticides is the result of a balance between uptake and translocation and the degree of those two processes will dictate the treatment effectiveness.

Lipophilicity is the most important property that regulates uptake and translocation of non-ionized pesticides. Pesticide mobility and lipophilicity are negatively correlated. In general, highly polar or highly lipophilic compounds are poorly translocated. The optimum uptake by roots and translocation to shoots occurs for pesticides of log*K*_ow_ values 1–3 (Bromilow and Chamberlain, 1995; Sicbaldi et al., 1997). Uptake and translocation of ionized pesticides within plant compartments (pH ranges from 5 to 8) are also affected by pH values while ion trapping is a well-studied mechanism of accumulation of weak acids in cytoplasm (Briggs et al., 1987; Chamberlain et al., 1998). The apoplastic and symplastic pathways have been proposed to explain the rationality of pesticide root uptake and translocation (Sicbaldi et al., 1997). In both cases, transmembrane movement of pesticides happens by taking advantage of passive, active, and facilitated diffusion, though ATP-powered pumps, channel proteins and transporters (uni-, anti-, and sym-porters). The movement of pesticides toward the top of the plant may occur in both the xylem and the phloem. Moreover, lateral transport has been observed in some cases. Nutrient and other carrier systems are usually involved in pesticides transportation across cell membranes and translocation within the plant (Chen et al., 2001; Xia et al., 2014).

## Storage, Metabolism, and Action of Soil Applied Pesticides

The fate of pesticides varies in different plant parts. Storage in cell organelle, metabolism, interaction with physiological, and biochemical processes, signaling and action are the main processes by which a pesticide interacts with the plant and target organism tissues.

The detoxification process of many pesticides carried out through conjugation by the plant constitutes such as glutathione, glucose, carbohydrates, amino acids, and glucuronic acid. The largest amounts of bound and conjugated pesticides are frequently stored close to the point of uptake and in tissues with intense metabolic activity (Norris, 1974). Pesticide storage in specific cell organelles (vacuole) can be achieved actively or passively through membranes. Both processes are reversible and translocation to other plant compartments may occur under different plant physiological conditions such as drought stress, phytohormones effect and nutrients cross talk (Schröder and Stampfl, 1999; Diekmann et al., 2004; Schröder et al., 2007).

Metabolism is nearly always a detoxification process of a pesticide for the target organism (plant, pathogen, and pest), but many metabolites are biologically active and may have physiological, ecological, and toxicological significance. However, in other cases metabolism can activate propesticides (e.g., indoxacarb, benomyl, benzobicyclon) and modify their effectiveness and fate (uptake and translocation) within plants (Jeschke, 2016).

Following the chemical pesticide revolution after the 1930s, multitude of agrochemical became available and scientists all over the world from industry, institutions, universities and registration authorities, focused their research on the clarification of the mechanism of their action on target sites of pests, weed and pathogens (efficacy) and on non-target organisms (toxicity). Today, more than 100 mechanisms of pesticides action have been revealed among the approximately 900 currently commercially available pesticides (Casida, 2009; Tomlin, 2009). In many cases, the initial proposed main mechanism of action was readjusted or new secondary site of action and biochemical or physiological effects were interpreted later on. Moreover, the acute, chronic, hypersensitive or delayed toxicity, of many legacy pesticides, to not-target organisms had been revealed after using them for decades. Most currently existing pesticides interact with a vital biochemical process of the target organisms. According to their chemical structure, herbicides, insecticides and fungicides suppress fundamental biosynthetic processes or deviate specific reactions. Most of pesticide target sites and respective inhibited biochemical processes are located or at least include biological membranes (**Table 1**). Biological membranes support numerous cell functions which are targeted by pesticides while simultaneously, the cell compartments affect the behavior of pesticides the most (permeability, translocation, and action of pesticides, signaling, interaction with root exudates and microbiome produced substances; **Table 1**). Moreover, genetic or epigenetic modifications on target organisms, leading to biochemical and physiological differences on biological membranes, are usually involved in the development of resistance mechanisms against pesticides (R4P Network, 2016).

**Table 1:** Examples of pesticides with membrane-related activity.

	Chemical class	Representative compound	Major target site	Membrane-related physiological function affected
Insecticides	Organophosphates	Chlorpyrifos	Acetylcholinesterase	Chemical transmission of nerve impulse to post-synaptic **membrane** of nervous system
	Carbamates	Oxamyl		
	Neonicotinoids	Imidacloprid	Nicotinic acetylcholine receptors (nAChR), agonist action on nAChR	nAChR subunits contains trans**membrane** domains, extracellular terminus and intracellular loop
	Spinosyns	Spinosad	Activation of nAChR	
	Nereistoxin analogs	Cartap	Blocks of nAChR	
	Cyclodiene	Endosulfan	GABA-gated chloride channels	GABA and glutamate receptors coupled to chloride channels are located at postsynaptic **membrane** of neuronal dendritic spine
	Phenylpyrazoles	Fipronil		
	Avermectins	Abamectin	Glutamate-gated chloride channels	
	Organochlorines	DDT	Voltage-gated sodium channel (vgsc)	Vgsc consists of a single polypeptide chain with four internally homologous domains each having six trans**membrane** helices located at the insect axonal **membrane** of nervous cells- organochlorines and pyrethroids binds to the vgsc and locks it in the open state
	Pyrethroids	Deltamethrin		
	Oxadiazine	Indoxacarb		
	METIs	Fenazaquin	Inhibit electron transport during ATP production	The electron transport chain is a series of cytochromes involved in the production of energy (ATP) located at mitochondrial **membrane**
	Microbial	*Bacillus Thuringiensis* proteins (δ-endotoxins)	Peritrophic membrane pore formation	Microbial disruption of midgut **membranes**
	Diamides	Chlorantraniliprole	Ryanodine receptor and release of Ca^2+^	Ryanodine receptors are a family of Ca^2+^ release channels located at the endoplasmic reticulum **membrane** and are responsible for the release of Ca^2+^
	Formamidine	Amitraz	Octapamine receptors	Octopamine receptors or G-protein coupled receptor have seven trans**membrane** domains located at insect nervous system and influencing multiple physiological events
	Benzoylphenylurea	Diflubenzuron	Chitin biosynthesis	chitin synthase are large trans**membrane** proteins
Herbicides	Triazines	Terbuthylazine	Photosynthesis inhibitor	D-1 quinone-binding protein of photosynthetic electron transport chain is located at thylakoid **membrane** of chloroplasts (Photosystem II receptor)
	Ureas	Linuron		
	Nitrile	Bromoxynil		
	Triketone	Mesotrione	Pigment inhibitors	Inhibit *p*-hydroxyphenyl pyruvate dioxygenase which is located at the thylakoid **membrane**
	Aryloxyphenoxypropionic	Fenoxaprop	Acetyl CoA carboxylase inhibitors	Acetyl CoA carboxylase is located at plastid **membranes**. Inhibition of fatty acid synthesis blocks the production of phospholipids used in building new **membranes** required for cell growth
	Sulfonylurea	Nicosulfuron	Amino acid synthesis inhibitors	Acetolactate synthase is located at chloroplast **membrane**
	Diphenyl ether	Acifluorfen	Protoporphyrinogen oxidase inhibitors-cell membrane disrupters	Protoporphyrinogen oxidase is located at thylakoid **membrane** of chloroplasts
	Bipyridylium	Paraquat	Photosystem I electron eiverter	Produce lipid hydroperoxides and destroy the integrity of cell **membranes**
	Chloroacetamide	Metolachlor	Long-chain Fatty Acid Inhibitors	Long-chain fatty acids are involved in many **membrane**-related biochemical responses of epidermis cells such as cell proliferation
	Thiocarbamate	EPTC		
Fungicides	Phosphorothiolates	Pyrazophos	Phospholipid biosynthesis	These fungicides are directly affect lipid synthesis and **membrane** permeability and integrity
	Aromatic hydrocarbons	Quintozene	Lipid peroxidation	
	Carbamates	Propamocarb	Cell membrane permeability fatty acids and phospholipid inhibitor	
	Microbial	Lipopeptides from *B.* Subtilis	Membrane integrity	Microbial disrupters of pathogen cell **membranes**
	Pyridine	Boscalid	Inhibit fungal respiration by blocking the succinate-dehydrogenase sites in the mitochondrial complex II	Succinate dehydrogenase is an enzyme complex, bound to the inner mitochondrial **membrane** - It is the only enzyme that participates in both the citric acid cycle and the electron transport chain
	Organotins	Fentin acetate	Inhibitors of oxidative phosphorylation, ATP synthase	ATP synthase is located at inner mitochondrial **membrane**
	Triazoles	Myclobutalin	C14-demethylase during sterol biosynthesis by inhibiting the mixed-function oxygenase cytochrome P450	All these fungicides targeting cell **membrane** integrity by inhibiting sterol formation
	Imidazoles	Imazalil		
	Pyrimidines	Fenarimol		
	Morpholines	Fenpropimorph	Δ14-Reductase and Δ8→ Δ7-isomerase in sterol biosynthesis	
	Phenylpyrroles	Fludioxonil	Mitogen-activated protein/histidine- Kinase in osmotic signal transduction	Kinases are located at cell **membranes** and influencing signal transduction
	Dicarboximides	Iprodione		

## Prospects and Challenges

Our knowledge concerning the fate of SPs within plant and target organisms is limited due to previous decades results, based mainly on experiments concerning their mode of action and the potential of using plants for phytoremediation purposes (Casida, 2011; Vymazal and Brezinova, 2015). Plants as meta-organisms create numerous new perspectives for pesticide science. Awareness of recently acquired insights related to the plant “metaphysiology,” rhizosphere, plant microbiome, and their interplay with pesticides should now be taken under consideration (Berg et al., 2015). The metabolism and morphology of plants, their microbiota and pesticides innately interact with each other and can contribute to the proper function of the holobiont. For many pesticides we do not yet have a complete picture of the mechanisms that underlie the pesticide uptake and traverse the plant root, delivery to target sites and storage, or detoxification processes (Hurtado et al., 2016). The recently acquired knowledge on drug delivery systems, studied nowadays in medicine, has not far attempted in pesticide uptake and delivery to target sites. Based on the increasingly available body of evidence discussed in this article, the use of nanopesticides combined with knowledge on membranes biochemistry can give new perspectives to next-generation SPs (Cho et al., 2008; Pan et al., 2012). Uptake and delivery of pesticides to various plants and target organisms’ organelles and biological membranes should not only be studied in relation to their mode of action but also in the light of metaorganismal interactions and chemical ecology (**Figure 1**). The side or collateral effects of SPs on plant “metaphysiology” and not only on their target sites is another interesting research task related to SPs. This kind of side effects are more obvious if we consider the hormone-like action of many pesticides and that membrane functions, apart from pesticides, are usually regulated by effectors, elicitors, and hormones (Gerbeau-Pissot et al., 2014). As mentioned above, mode of action of most currently used pesticides involves or interacts with biological membranes. Thus, pesticide translocation and action should be studied in combination with transmembrane crosstalk and signal transduction pathways among membranous cell compartments (**Figure 1**). The appropriate structural modification of the active ingredient, the synthesis of propesticides and plant extract analogs, the development of nanopesticides and pesticide delivery systems, the introduction of new formulation for the combined application of pesticides with biocontrol agents (e.g., PGPR and pesticides in seed coating technology) are the most important concepts to design modern agrochemicals. Target-specific delivery and activation of propesticides, biopesticides and biotech-pesticides exactly at the target side or only in the presence of pest or pathogen could reduce largely the application dose of pesticides and minimize the adverse effects. Moreover, structure-optimized effects on transporters, transmembrane proteins, and enzymes could be the substantial functions of the next-generation SPs (Jeschke, 2016). Polymer-lipid and other hybrid nanomaterials possessing different material properties such as hydrophobicity and water solubility should also be studied for SPs (Byrappa et al., 2008; Wu et al., 2012). Although the effect of soil applied pesticides on soil microbial community structure and rhizo-epiphytic microorganisms has been extensively studied, the effects on endophytic consortium and seed microbiome have to be studied further (Karpouzas et al., 2016; Rousidou et al., 2016). Knowledge on the interaction of SPs with endophytic microorganisms and their enzymatic activity can improve the efficacy of existing or new pesticides. Further studies are needed to better understand the interplay of simultaneous application of pesticides, biological agents (e.g., PGPR, transfer of bespoke core-microbiome) and other compounds affecting biological membranes and target organisms’ physiology, biochemistry, ecology, and ethology (metabolites, elicitors, semiochemicals, signal transducers, hormones, growth regulators, and nutrients). The application of innovative instrumental analysis in combination with bioinformatics and metabolomics can be used to study the reciprocal interaction between SPs and plant microbiome.

## Concluding Remarks

All the phyto-microbial effects listed above open new windows for the next-generation SPs. A “scientific dialog” and research are required in order to reclaim all acquired knowledge and take advantage of progress in sciences related to pesticides, pharmaceutical, xenobiotics, medicine, plant physiology and signaling, microorganisms and pests. We should therefore try to study the physiological responses of target organisms to pesticides in a wider context. Pesticides will continue to play an important role in plant protection for the next decades under the concept of integrated pest management. Consequently, scientific advances discussed above could give the opportunity to deal with thoroughly the plant health and lustily instead of plant protection. Moreover, the expansion of the increasing pesticides related knowledge, which is usually acquired at a single organism scale (plant, pathogen, pests), to the agroecosystem scale is the fundamental challenge for the next-generation pesticides and plant hygiene in general.

## Author Contributions

ZV as sole author designed, developed, and wrote the ideas and content presented in this manuscript.

## Conflict of Interest Statement

The author declares that the research was conducted in the absence of any commercial or financial relationships that could be construed as a potential conflict of interest.
